# Sic Transit Gloria Mundi: A Mathematical Theory of Popularity Waves Based on a SIIRR Model of Epidemic Spread

**DOI:** 10.3390/e27060611

**Published:** 2025-06-09

**Authors:** Nikolay K. Vitanov, Zlatinka I. Dimitrova

**Affiliations:** Institute of Mechanics, Bulgarian Academy of Sciences, Acad. G. Bonchev Str., Bl. 4, 1113 Sofia, Bulgaria; zdim@imbm.bas.bg

**Keywords:** SIIRR model of epidemics, SIR model of epidemics, nonlinear differential equations, exact solutions, waves of popularity, suppression of popularity, shift of the peak of popularity, window of dominance

## Abstract

We discuss the spread of epidemics caused by two viruses which cannot infect the same individual at the same time. The mathematical modeling of this epidemic leads to a kind of SIIRR model with two groups of infected individuals and two groups of recovered individuals. An additional assumption is that after recovering from one of the viruses, the individual cannot be infected by the other virus. The mathematical model consists of five equations which can be reduced to a system of three differential equations for the susceptible and for the recovered individuals. This system has analytical solutions for the case when one of the viruses infects many more individuals than the other virus. Cases which are more complicated than this one can be studied numerically. The theory is applied to the study of waves of popularity of an individual/groups of individuals or of an idea/group of ideas in the case of the presence of two opposite opinions about the popularity of the corresponding individual/group of individuals or idea/group of ideas. We consider two cases for the initial values of the infected individuals: (a) the initial value of the individuals infected with one of the viruses is much larger than the initial values of the individuals infected by the second virus, and (b) the two initial values of the infected individuals are the same. The following effects connected to the evolution of the numbers of infected individuals are observed: 1. arising of bell-shaped profiles of the numbers of infected individuals; 2. suppression of popularity; 3. faster increase–faster decrease effect for the peaks of the bell-shaped profiles; 4. peak shift in the time; 5. effect of forgetting; 6. window of dominance; 7. short-term win–long-term loss effect; 8. effect of the single peak. The proposed SIIRR model is used to build a mathematical theory of popularity waves where a person or idea can have positive and negative popularity at the same time and these popularities evolve with time.

## 1. Introduction

Complex systems of different scales are numerous in the human and animal worlds, for example, research groups, social networks, economic systems, etc. [1,2,3,4,5,6]. The nonlinearity of these complex systems [7,8,9] complicates their study. Such systems are usually modeled by nonlinear differential equations. In these cases, one of the goals is to obtain exact solutions to these equations if possible. These solutions can be very useful, as we can easily study the relationships among the parameters of the complex system of interest.

In this article, we introduce a nonlinear model of the spread of epidemic waves (the SIIRR model of epidemics). We discuss an analytical solution connected to this model. In addition, we discuss numerical solutions of the model equations which allow us to exploit the influence of the model parameters on the evolution of the solutions. Many models exist for the spread of epidemics. One of the most basic of these models is the SIR model for describing the temporal dynamics of an infectious disease in a population [10,11,12,13,14,15,16,17,18,19,20,21,22,23,24,25,26]). The model compartmentalizes people into one of three categories: (i) susceptible to the disease; (ii) those who are currently infectious, and (iii) those who have recovered (with immunity). The SIR model is a set of three equations that describes the number of individuals in each compartment at every point in time. Epidemic models can also be applied for the description of other processes such as the spread of ideas (for overviews, see [4,27]). We also note the use of epidemic models for the study of COVID-19 spreading [28,29,30,31,32,33,34,35,36,37,38,39,40,41,42] as well as numerical methods for obtaining solutions for such models [43,44].

The text below is organized as follows. In Section 2, we introduce the SIIRR model. In Section 3, we find an exact solution to a system of equations obtained from the model equations with specific conditions and discuss the stability of the solutions to the model. Section 4 is devoted to the numerical study of the model system of equations for two different cases of values of the initial number of infected individuals: (a) $I_{1}\left(0\right)>>I_{2}\left(0\right)$ and (b) $I_{1}\left(0\right)=I_{2}\left(0\right)$. Several concluding remarks are given in Section 5.

## 2. The SIIRR Model of Spread of Two Viruses in a Population

The classic SIR model of epidemic spread [45] considers an epidemic in a population which is divided into three groups: susceptible individuals—*S*; infected individuals—*I*; and recovered individuals—*R*. The model equations for the time evolution of the numbers of individuals from these three groups are:

(1) $$\begin{array}{ccc} \frac{dS}{dt} & = & -\frac{\tau }{N}SI,\\ \frac{dI}{dt} & = & \frac{\tau }{N}SI-\rho I,\\ \frac{dR}{dt} & = & \rho I. \end{array}$$

In (1), τ is the transmission rate and ρ is the recovery rate. These rates are assumed to be constants. From (1), we have the relationship

(2) N=S+I+R.

*N* is the total population which is assumed to be constant.

We extend this model by means of the following assumptions. We assume that two kinds of infections are possible. The individuals who are infected by the first infection cannot be infected by the second infection. We will denote these infected individuals by $I_{1}$ and $I_{2}$. These two kinds of infected individuals lead to two kinds of recovered individuals: $R_{1}$ and $R_{2}$. The model equations are as follows:

(3) $$\begin{array}{ccc} \frac{dS}{dt} & = & -\frac{\tau_{1}}{N}SI_{1}-\frac{\tau_{2}}{N}SI_{2},\\ \frac{dI_{1}}{dt} & = & \frac{\tau_{1}}{N}SI_{1}-\rho_{1}I_{1},\\ \frac{dI_{2}}{dt} & = & \frac{\tau_{2}}{N}SI_{2}-\rho_{2}I_{2},\\ \frac{dR_{1}}{dt} & = & \rho_{1}I_{1},\\ \frac{dR_{2}}{dt} & = & \rho_{2}I_{2}. \end{array}$$

The model is specially chosen for application in the area of mathematical social dynamics for the description of the process of evolution of popularity waves. There are two waves: the wave of positive popularity and the wave of negative popularity. We assume that the increase and decrease in popularity are processes similar to the spread of an infection. We have two kinds of popularity, and because of this, we need two populations of infected individuals. The decrease in popularity can happen at different intensities and because of this, we have two populations of recovered individuals. Another reason to count the populations of recovered individuals separately is that the past may influence the future: the parameters of the next wave of popularity which affect the population may differ for the groups of individuals who contributed to the positive or negative popularity in a previous popularity wave.

We note that the model (3) is a kind of multi-strain epidemic model [46,47,48,49,50,51,52,53,54,55,56]. The model (3) is of the class of two-strain models. Such models are also often studied in epidemiology [57,58,59,60,61]. We note that many of the above-mentioned models have a single population of recovered individuals [47,49,57,58,60].

In (3) $\tau_{1,2}$ are the transmission rates and $\rho_{1,2}$ are the recovery rates. These rates are assumed to be constants. From (3) we have the relationship

(4) $$N=S+I_{1}+I_{2}+R_{1}+R_{2}.$$

The fixed points of the model (3) are obtained by setting the derivatives in (3) to 0. The fixed points are $\left(S^{*},I_{1}^{*},I_{2}^{*},R_{1}^{*},R_{2}^{*}\right)$ = $\left(S^{*},0,0,R_{1}^{*},R_{2}^{*}\right)$ where $S^{*},R_{1}^{*},R_{2}^{*}$ can have any admissible value. From (8) we have $R_{2}^{*}=N-S^{*}-R_{1}^{*}$. Then the fixed points can be written as $\left(S^{*},I_{1}^{*},I_{2}^{*},R_{1}^{*},R_{2}^{*}\right)$ = $\left(S^{*},0,0,R_{1}^{*},N-S^{*}-R_{1}^{*}\right)$ which is a two-parameter family of points.

For the linear stability of the fixed points, we obtain the following result for det(J−λI) where *J* is the Jacobian of the system of Equation (3) and *I* is the unit matrix.

(5) $$\begin{array}{c} \det\left(J-\lambda I\right)=\lambda^{2}\{\frac{\tau_{2}^{2}I_{2}S}{N^{2}}\left(\frac{\tau_{1}S}{N}-\rho_{1}-\lambda_{1}\right)+\\ \left(\frac{\tau_{2}S}{N}-\rho_{2}-\lambda \right)\left[\left(-\frac{\tau_{1}I_{1}}{N}-\frac{\tau_{2}I_{2}}{N}-\lambda \right)\left(\frac{\tau_{1}}{N}S-\rho_{1}-\lambda \right)+\frac{\tau_{1}^{2}SI_{1}}{N^{2}}\right]\}. \end{array}$$

For the fixed points $\left(S^{*},0,0,R_{1}^{*},N-S^{*}-R_{1}^{*}\right)$, this leads to the following values for λ

(6) $$\lambda_{1,2,3}=0;\;\;\lambda_{4}=\frac{\tau_{2}}{N}S^{*}-\rho_{2};\;\;\lambda_{5}=\frac{\tau_{1}}{N}S^{*}-\rho_{1}.$$

Thus, it follows from the linear stability analysis that for $S^{*}\leq N\frac{\rho_{2}}{\tau_{2}}$ and for $S^{*}\leq N\frac{\rho_{1}}{\tau_{1}}$, we have marginal stability of the corresponding fixed point. Otherwise, the fixed point is unstable. Then, for a large enough $S^{*}$, the fixed point will be unstable.

The SIR model is a specific case of the considered SIIRR model. This specific case is obtained when $R_{2}=0$. The same specific case is also obtained when $R_{1}=0$. We note that there are analytical solutions connected to the SIR model as well as to similar models such as the SEIR model of epidemic spread. These solutions can be obtained by a methodology called SEsM (Simple Equations Method) [62,63,64,65,66].

Next, we will reduce the system of Equation (3) to a system of equations for the quantities *S*, $R_{1}$, and $R_{2}$. First of all, we obtain from the last two equations of the system (3)

(7) $$\begin{array}{c} I_{1}=\frac{1}{\rho_{1}}\frac{dR_{1}}{dt},\\ I_{2}=\frac{1}{\rho_{2}}\frac{dR_{2}}{dt}. \end{array}$$

In addition, from (4) we obtain

(8) $$\begin{array}{c} I_{1}=N-S-I_{2}-R_{1}-R_{2},\\ I_{2}=N-S-I_{1}-R_{1}-R_{2}. \end{array}$$

We substitute (8) in (7). The result is

(9) $$\begin{array}{c} \frac{1}{\rho_{1}}\frac{dR_{1}}{dt}=N-S-I_{2}-R_{1}-R_{2},\\ \frac{1}{\rho_{2}}\frac{dR_{2}}{dt}=N-S-I_{1}-R_{1}-R_{2}. \end{array}$$

In (9), we substitute $I_{1}$ and $I_{2}$ from (7). Thus, we arrive at the equation

(10) $$\frac{1}{\rho_{1}}\frac{dR_{1}}{dt}+\frac{1}{\rho_{2}}\frac{dR_{2}}{dt}=N-R_{1}-R_{2}-S.$$

We need two additional equations for *S*, $R_{1}$ and $R_{2}$. The first of these equations is obtained by substitution of (7) into the first equation of (3). The result is

(11) $$\frac{1}{S}\frac{dS}{dt}=-\frac{1}{N}\left(\frac{\tau_{1}}{\rho_{1}}\frac{dR_{1}}{dt}+\frac{\tau_{2}}{\rho_{2}}\frac{dR_{2}}{dt}\right).$$

The third equation for the system of equations for $R_{1}$, $R_{2}$, and *S* can be obtained as follows. From the fourth equation of (3), we obtain

(12) $$\frac{d^{2}R_{1}}{dt^{2}}=\rho_{1}\frac{dI_{1}}{dt}.$$

We substitute here $\frac{dI_{1}}{dt}$ from the second equation of (3). The result is

(13) $$\frac{d^{2}R_{1}}{dt^{2}}=\rho_{1}\left(\frac{\tau_{1}}{N}SI_{1}-\rho_{1}I_{1}\right).$$

In (13), we substitute $I_{1}$ from (7). The result is

(14) $$\frac{d^{2}R_{1}}{dt^{2}}=\rho_{1}\frac{dR_{1}}{dt}\left(\frac{\tau_{1}}{\rho_{1}N}S-1\right)$$

Thus, we have the system of three Equations (10), (11) and (14) for *S*, $R_{1}$ and $R_{2}$. Let us note that this system contains the system of two equations for *S* and *R* or the SIR model of epidemic spread. In order to show this, let us set $I_{2}=0$ and $R_{2}=0$. Then (3) is reduced to the model system of equations for the SIR model. We note that the system of equations for the SIR model can be reduced to a single equation for *R* as follows.

## 3. An Exact Solution to Equations Connected to the Model Equations for the SIIRR Model

We can obtain an exact solution to an equation which describes a specific case of the SIIRR model. In order to do this, we will proceed in analogy with the procedure of obtaining an exact solution to a sequence of equations connected to the SIR model of epidemic spread by means of the SEsM [67]. We start with the equations for the SIIRR model, (10), (11) and (14). From (11) we obtain the following for S(t) by means of an integration:

(15) $$S=S\left(0\right)\exp\left[-\frac{\tau_{1}}{\rho_{1}N}\left(R_{1}-R_{1}\left(0\right)\right)\right]\exp\left[-\frac{\tau_{2}}{\rho_{2}N}\left(R_{2}-R_{2}\left(0\right)\right)\right],$$

where S(0), $R_{1}\left(0\right)$ and $R_{2}\left(0\right)$ are constants of integration. We have to deal with exponential nonlinearities. We assume that $R_{1,2}\left(0\right)=0$: there are no recovered individuals at t=0. Then, we discuss the case where $\frac{\tau_{1}R_{1}}{\rho_{1}N}<<1$ and $\frac{\tau_{2}R_{2}}{\rho_{2}N}<<1$. This will allow us to represent the two exponents in (15) as Taylor series. In addition, we discuss the case of “small $R_{2}$ and larger $R_{1}$” (in the same way, we can consider the case of “small $R_{1}$ and larger $R_{2}$” ). In this case, we assume that $\frac{\tau_{2}R_{2}}{\rho_{2}N}$ is so small that $\exp\left[-\frac{\tau_{2}}{\rho_{2}N}R_{2}\right]$ in (15) is practically equal to 1. Then (15) is reduced to

(16) $$S\approx S\left(0\right)\exp\left[-\frac{\tau_{1}}{\rho_{1}N}R_{1}\right]\approx S\left(0\right)\sum_{j=0}^{M}{\left(-\frac{\tau_{1}R_{1}}{\rho_{1}N}\right)}^{j}.$$

Thus, from (10) and (14), we obtain a system of two equations which can have exact analytical solutions. This system is as follows. The substitution of (16) into (14) leads to the equation

(17) $$\frac{dR_{1}}{dt}=C+\frac{\tau_{1}S\left(0\right)}{N}R_{1}-\rho_{1}R_{1}+\sum_{j=1}^{M}\frac{\tau_{1}S\left(0\right)}{(j+1)N}{\left(\frac{-\tau_{1}}{\rho_{1}N}\right)}^{j}R_{1}^{j+1},$$

where *C* is a constant of integration. We set

(18) $$\alpha_{0}=C,\;\;\alpha_{1}=\frac{\tau_{1}S\left(0\right)}{N}-\rho_{1},\;\;\alpha_{j+1}=\frac{\tau_{1}S\left(0\right)}{(j+1)N}{\left(\frac{-\tau_{1}}{\rho_{1}N}\right)}^{j},\;\;j=1,2,\ldots$$

Then, Equation (17) is reduced to the equation

(19) $$\frac{dR_{1}}{dt}=\sum_{k=0}^{M+1}\alpha_{k}R_{1}^{k}.$$

Let us assume that we have an exact solution to this equation. Then (10) is a linear equation with respect to $R_{2}$. This equation has the form

(20) $$\begin{array}{c} \frac{dR_{2}}{dt}+\rho_{2}R_{2}=\left(-\frac{\alpha_{0}\rho_{2}}{\rho_{1}}+\rho_{2}N-\rho_{2}S\left(0\right)\right)+\left(-\frac{\rho_{2}\alpha_{1}}{\rho_{1}}-\rho_{2}-\frac{\rho_{2}S\left(0\right)\tau_{1}}{\rho_{1}N}\right)R_{1}+\\ \sum_{j=1}^{M}\left(-\frac{\rho_{2}}{\rho_{1}}\alpha_{j+1}\right)R_{1}^{j+1}+\sum_{j=1}^{M}\left[-\rho_{2}S\left(0\right){\left(\frac{-\tau_{1}}{\rho_{1}N}\right)}^{j}\right]R_{1}^{j}. \end{array}$$

We set

(21) $$\begin{array}{ccc} p & = & \rho_{2};\\ \beta_{0} & = & -\frac{\alpha_{0}\rho_{2}}{\rho_{1}}+\rho_{2}N-\rho_{2}S\left(0\right);\\ \beta_{1} & = & -\frac{\rho_{2}\alpha_{1}}{\rho_{1}}-\rho_{2}-\frac{\rho_{2}S\left(0\right)\tau_{1}}{\rho_{1}N};\\ \beta_{j+1} & = & -\frac{\rho_{2}}{\rho_{1}}\alpha_{j+1};\\ \gamma_{j} & = & -\rho_{2}S\left(0\right){\left(\frac{-\tau_{1}}{\rho_{1}N}\right)}^{j};\\ q(t) & = & \beta_{0}+\beta_{1}R_{1}+\sum_{j=1}^{M}\beta_{j+1}R_{1}^{j+1}+\sum_{j=1}^{M}\gamma_{j}R_{1}^{j}. \end{array}$$

Then, (21) becomes

(22) $$\frac{dR_{2}}{dt}+pR_{2}=q\left(t\right).$$

The solution of (22) is

(23) $$R_{2}=\exp\left(-pt\right)\left\{D+\int dt\;[q(t)\exp(pt)]\right\},$$

where *D* is a constant of integration.

Here, we discuss the most simple case: M=1. Then (19) becomes

(24) $$\frac{dR_{1}}{dt}=\alpha_{2}R_{1}^{2}+\alpha_{1}R_{1}+\alpha_{0}.$$

(24) is an equation of the Riccati kind. For this equation, we know the specific solution

(25) $$R_{1}\left(t\right)=-\frac{\alpha_{1}}{2\alpha_{2}}-\frac{\theta }{2\alpha_{2}}\tanh\left[\frac{\theta (t+V)}{2}\right],$$

where $\theta^{2}=\alpha_{1}^{2}-4\alpha_{0}\alpha_{2}>0$ and *V* is a constant of integration. On the basis of the specific solution (25) of (24), we can write the general solution of (24) as $R=-\frac{a\alpha_{1}}{2\alpha_{2}}-\frac{\theta }{2\alpha_{2}}\tanh\left[\frac{\theta (x+V)}{2}\right]+\frac{D}{v}$ where *W* is a constant and v(t) is the solution of the linear differential equation

(26) $$\frac{dv}{dx}-\theta \tanh\left[\frac{\theta (t+V)}{2}\right]v=-\alpha_{2}W$$

The solution of (26) is

(27) $$v=\cosh^{2}\left[\frac{\theta (t+V}{2}\right]\left\{E-\frac{2\alpha_{2}W}{\theta }\tanh\left[\frac{\theta (t+V)}{2}\right]\right\},$$

where *E* is a constant of integration. Then, the general solution of Equation (24) is

(28) $$R_{1}\left(t\right)=-\frac{\alpha_{1}}{2\alpha_{2}}-\frac{\theta }{2\alpha_{2}}\tanh\left[\frac{\theta (t+V)}{2}\right]+\frac{W}{\cosh^{2}\left[\frac{\theta (t+V)}{2}\right]\left\{E-\frac{2\alpha_{2}W}{\theta }\tanh\left[\frac{\theta (t+V)}{2}\right]\right\}}.$$

Let us now obtain the form of several solutions to the system of Equations (19) and (20). For M=1, we have for $R_{1}$ a solution (28) to the corresponding Equation (19). We have to substitute this into (20) in order to obtain the corresponding solution for $R_{2}\left(t\right)$. In order to obtain an analytical result, we will consider the specific case W=0 and $\rho_{2}=\rho_{1}$. Then the solution for $R_{2}$ is

(29) $$\begin{array}{c} R_{2}\left(t\right)=D\exp\left(-\rho_{1}t\right)-\frac{\theta }{\alpha_{2}\left\{1+\exp\left[\theta \left(t+V\right)\right]\right\}}+\frac{1}{4\rho_{2}\alpha_{2}^{2}}[-\alpha_{1}^{2}\alpha_{2}-2\alpha_{1}\alpha_{2}\left(-\alpha_{1}-\rho_{2}-\frac{S_{0}\tau_{1}}{N}\right)-\\ \frac{2\alpha_{1}\alpha_{2}S_{0}\tau_{1}}{N}-2\alpha_{1}\alpha_{2}\theta +4\alpha_{2}^{2}\left(\rho_{2}N-\rho_{2}S_{0}-\alpha_{0}\right)-2\alpha_{2}\left(-\alpha_{1}-\rho_{2}-\frac{S_{0}\tau_{1}}{N}\right)\theta -\frac{2\alpha_{2}S_{0}\tau_{1}\theta }{N}-\alpha_{2}\theta^{2}]. \end{array}$$

The constants of integration *V* and *D* are obtained from the requirements $R_{1}\left(0\right)=R_{2}\left(0\right)=0$. The relationships are as follows:

(30) $$\begin{array}{c} D=S\left(0\right)-N;\;\;\;V=\frac{2\tanh^{-1}\left[\frac{\rho_{2}N-\tau_{1}S\left(0\right)}{N{\left(\frac{N^{2}\rho_{2}^{3}-2NS\left(0\right)\rho_{2}^{2}\tau_{1}+S{\left(0\right)}^{2}\rho_{2}\tau_{1}^{2}+2CS\left(0\right)\tau_{1}^{2}}{\rho_{2}N^{2}}\right)}^{1/2}}\right]}{{\left(\frac{N^{2}\rho_{2}^{3}-2NS\left(0\right)\rho_{2}^{2}\tau_{1}+S{\left(0\right)}^{2}\rho_{2}\tau_{1}^{2}+2CS\left(0\right)\tau_{1}^{2}}{\rho_{2}N^{2}}\right)}^{1/2}}. \end{array}$$

In addition,

$$\theta ={\left(\frac{N^{2}\rho_{2}^{3}-2NS\left(0\right)\rho_{2}^{2}\tau_{1}+S{\left(0\right)}^{2}\rho_{2}\tau_{1}^{2}+2CS\left(0\right)\tau_{1}^{2}}{\rho_{2}N^{2}}\right)}^{1/2}.$$

From here, we can calculate the numbers of infected $I_{1}$ and $I_{2}$

(31) $$\begin{array}{c} I_{1}=\frac{1}{\rho_{1}}\frac{dR_{1}}{dt}=-\frac{\theta^{2}}{4\rho_{1}\alpha_{2}\cosh^{2}\left[\frac{\theta (t+V)}{2}\right]}. \end{array}$$

(32) $$\begin{array}{c} I_{2}=\frac{1}{\rho_{2}}\frac{dR_{2}}{dt}=\frac{1}{\rho_{2}}\left[-\rho_{1}D\exp\left(-\rho_{1}t\right)+\frac{\theta^{2}\exp\left[\theta \left(t+V\right)\right]}{\alpha_{2}{\left\{1+\exp\left[\theta \left(t+V\right)\right]\right\}}^{2}}\right]. \end{array}$$

(31) describes a bell-shaped profile and (32) corresponds to monotonic decay of the number of infected $I_{2}$. We will observe such kinds of solutions in the numerical study of the model equations in Section 4.

## 4. Several Results from the Numerical Study of the Model Equations

We will discuss two specific cases with respect to the initial values of $I_{1,2}$. The first specific case is when $I_{1}\left(0\right)>>I_{2}\left(0\right)$ (because of the symmetry of the model equations, the results will be the same for the case $I_{1}\left(0\right)<<I_{2}\left(0\right)$). The second specific case will be when $I_{1}\left(0\right)$ and $I_{2}\left(0\right)$ have the same values.

### 4.1. $I_{1}\left(0\right)>>I_{2}\left(0\right)$

**Figure 1:**
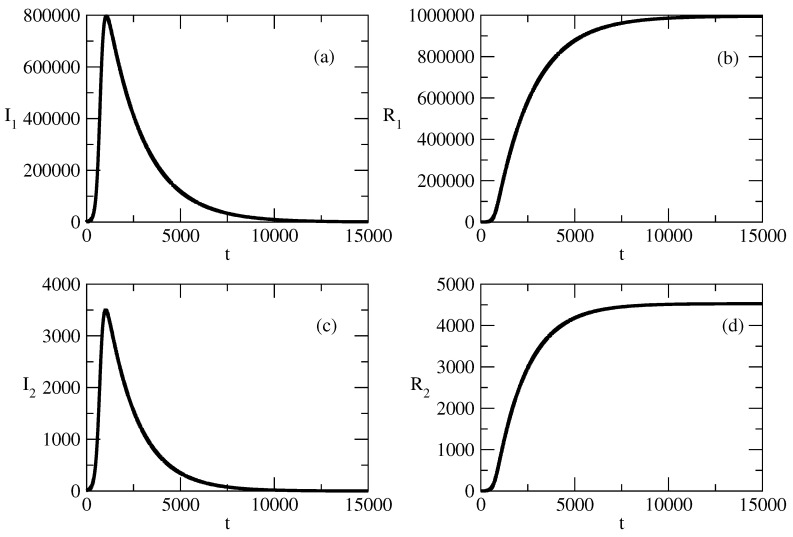
Basic solution for the studies for the case $I_{1}\left(0\right)>>I_{2}\left(0\right)$. (**a**) $I_{1}$. (**b**) $I_{2}$. (**c**) $R_{1}$. (**d**) $R_{2}$. The values of the parameters are as follows: $I_{1}\left(0\right)=1000$. $I_{2}\left(0\right)=10$. S(0)=998,990. $\tau_{1}=0.01$. $\tau_{2}=0.009$. $\rho_{1}=0.0005$. $\rho_{2}=0.0006$.

Figure 1 shows the basic solution for this study. For this solution, we choose $N=S+I_{1}+I_{2}=1,000,000$, and in addition, $I_{1}\left(0\right)$ is 100 times larger than $I_{2}$. We observe a large wave of $I_{1}$ and a much smaller wave of $I_{2}$. The peak of the wave of $I_{2}$ is slightly earlier than the peak of the wave of $I_{1}$. Over time, $I_{1,2}$ tends to 0 and the number of recovered individuals increases to *N*. We note that for the selected basic wave for our study, $\tau_{1}$ is slightly larger than $\tau_{2}$ and $\rho_{1}$ is slightly smaller than $\rho_{2}$.

The following figures show the influence of the parameters of the model on this basic solution.

**Figure 2:**
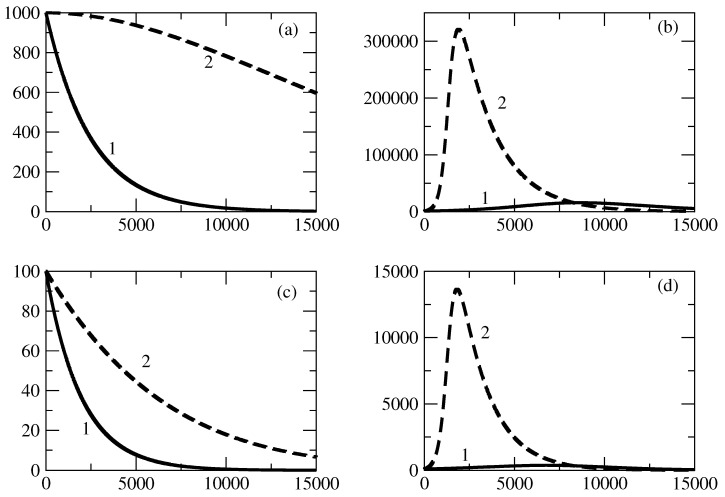
Influence of the value of S(0) on the profiles of $I_{1}$ and $I_{2}$ for the basic solution shown in Figure 1. (**a**) Line 1: $I_{1}$ for S(0)=10,000. Line 2: $I_{1}$ for S(0)=50,000. (**b**) Line 1: $I_{1}$ for S(0)=99,000. Line 2: $I_{1}$ for S(0)=500,000. (**c**) Line 1: $I_{2}$ for S(0)=10,000. Line 2: $I_{2}$ for S(0)=50,000. (**d**) Line 1: $I_{2}$ for S(0)=99,000. Line 2: $I_{2}$ for S(0)=500,000. The values of the parameters are as follows: $I_{1}\left(0\right)=1000$. $I_{2}\left(0\right)=10$. $\tau_{1}=0.01$. $\tau_{2}=0.009$. $\rho_{1}=0.0005$. $\rho_{2}=0.0006$.

Figure 2 shows the influence of the value of S(0) on the profiles of $I_{1,2}$. Figure 2a shows that for the case of small values of S(0), there is a monotonic decrease in $I_{1}$. For larger S(0), the profile of $I_{1}$ becomes bell-shaped, as can be seen in Figure 2b. Similar is the situation with the profile of $I_{2}$. For small values of S(0), we observe a monotonic decrease, and for larger values of S(0), the profile of $I_{2}$ becomes bell-shaped.

There is an interesting feature connected to the profiles of $I_{1,2}$. For the bell-shaped kind of profiles (Figure 2b,d), we observe faster motion to the peak for an increasing value of S(0). Then a fast decrease follows, and at large times, the profiles are closer to 0 when S(0) is larger.

Finally, we observe that for increasing S(0) for the case of a bell-shaped profile of the studied solution, the peak for $I_{2}$ is reached slightly sooner than the peak for $I_{1}$ and the initial ration $I_{1}\left(0\right)/I_{2}\left(0\right)=100$ is reduced to a value of about 30 in the area of the peaks.

**Figure 3:**
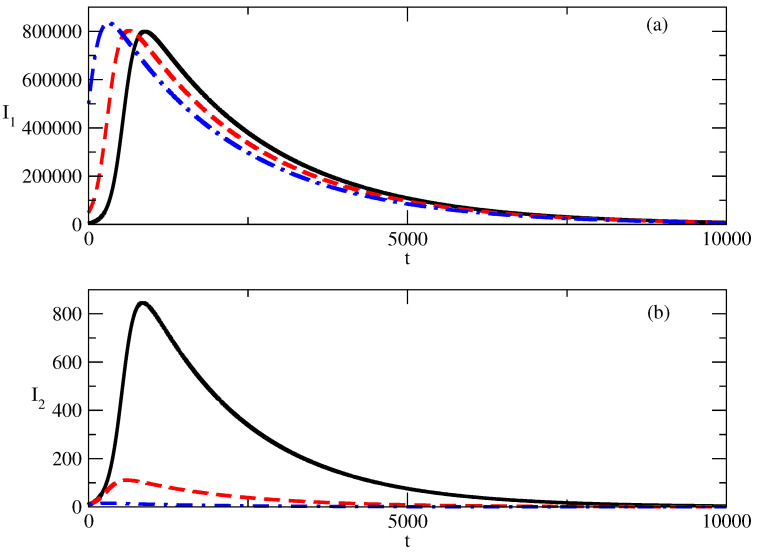
Influence of the value of $I_{1}\left(0\right)$ on the profiles of $I_{1}$ and $I_{2}$ for the basic solution shown in Figure 1. (**a**) Solid line: $I_{1}$ for $I_{1}\left(0\right)=5000$. Dashed line: $I_{1}$ for $I_{1}\left(0\right)=10,000$. Dot-dashed line: $I_{1}$ for $I_{1}\left(0\right)=5,000,000$. (**b**) Solid line: $I_{2}$ for $I_{1}\left(0\right)=5000$. Dashed line: $I_{2}$ for $I_{1}\left(0\right)=10,000$. Dot-dashed line: $I_{2}$ for $I_{1}\left(0\right)=5,000,000$. The values of the parameters are as follows: $I_{2}\left(0\right)=10$. $\tau_{1}=0.01$. $\tau_{2}=0.009$. $\rho_{1}=0.0005$. $\rho_{2}=0.0006$.

Figure 3 shows the influence of the initial value $I_{1}\left(0\right)$ of the first group of infected individuals on the profiles of the curves for $I_{1}\left(t\right)$ and $I_{2}\left(t\right)$. We note that the increase in $I_{1}\left(0\right)$ leads to the effect “Faster increase–faster decrease”: $I_{1}\left(t\right)$ reaches its peak value earlier in time and then decreases faster in comparison to the case where $I_{1}\left(0\right)$ has a smaller value. One also observes an increase in the value of the peak of $I_{1}\left(t\right)$ with an increase in $I_{1}\left(0\right)$.

Figure 3b shows the influence of the increase in the value of $I_{1}\left(0\right)$ on the value of $I_{2}\left(t\right)$. We note that $I_{2}\left(0\right)$ has the same value for all three cases shown in the figure. We observe two effects:

1. Suppression of the peak of $I_{2}\left(t\right)$ with an increase in $I_{1}\left(0\right)$. The peak of $I_{2}$ may even vanish.
2. The smaller peak of $I_{2}\left(t\right)$ occurs faster with an increasing value of $I_{2}\left(t\right)$.

**Figure 4:**
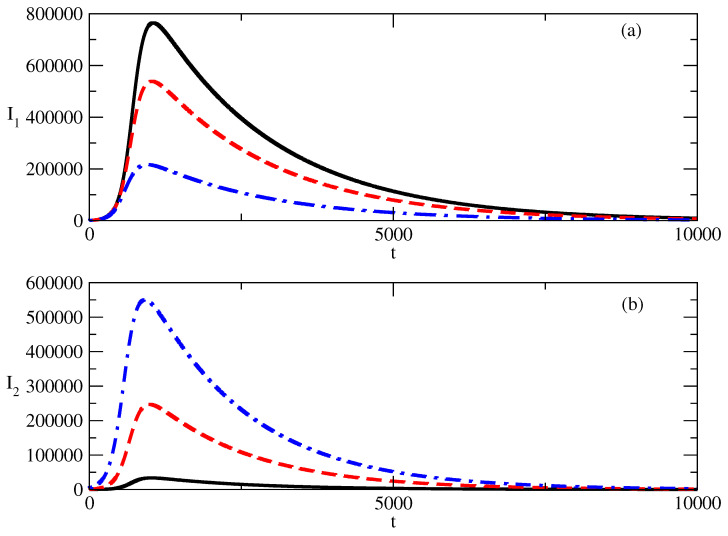
Influence of the value of $I_{2}\left(0\right)$ on the profiles of $I_{1}$ and $I_{2}$ for the basic solution shown in Figure 1. (**a**) Solid line: $I_{1}$ for $I_{2}\left(0\right)=100$. Dashed line: $I_{1}$ for $I_{2}\left(0\right)=1000$. Dot-dashed line: $I_{1}$ for $I_{2}\left(0\right)=5000$. (**b**) Solid line: $I_{2}$ for $I_{2}\left(0\right)=100$. Dashed line: $I_{2}$ for $I_{2}\left(0\right)=1000$. Dot-dashed line: $I_{2}$ for $I_{2}\left(0\right)=5000$. The values of the parameters are as follows: $I_{1}\left(0\right)=1000$. $\tau_{1}=0.01$. $\tau_{2}=0.009$. $\rho_{1}=0.0005$. $\rho_{2}=0.0006$.

Figure 4 shows the influence of the initial values of $I_{2}\left(0\right)$ on the numbers $I_{1,2}$ of infected individuals. Figure 4a shows that the increase in the initial value of $I_{2}$ suppresses the bell-shaped curve for $I_{1}\left(t\right)$. This suppression is accompanied by the shifting of the peak of $I_{1}$ to the region of earlier times. The opposite is observed in Figure 4b: the increase in the initial value of $I_{2}\left(0\right)$ leads to a larger peak for the $I_{2}(t)$ curve, and the decrease in $I_{2}$ after the peak is slower.

**Figure 5:**
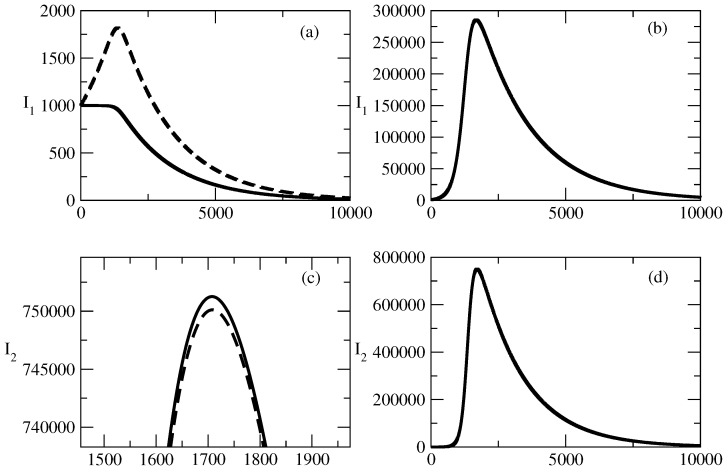
Influence of the value of $\tau_{1}$ on the profiles of $I_{1}$ and $I_{2}$ for the basic solution shown in Figure 1. (**a**) Solid line: $\tau_{1}=0.0005$. Dashed line: $\tau_{1}=0.001$. (**b**) $\tau_{1}=0.005$. (**c**) Solid line: $\tau_{1}=0.0005$. Dashed line: $\tau_{1}=0.001$. (**d**) $\tau_{1}=0.005$. The values of the parameters are as follows: $\tau_{2}=0.009$. $\rho_{1}=0.0005$. $\rho_{2}=0.0006$.

Figure 5 shows the influence of the change in the value of the parameter $\tau_{1}$ (the transmission rate for the population of infected individuals $I_{1}$) on the evolution of $I_{1}\left(t\right)$ and $I_{2}\left(t\right)$. Figure 5a shows the formation of the bell-shaped profile with an increase in the value of $\tau_{1}$. A further increase in the value of $\tau_{1}$ leads to a well-formed profile and a large peak, as shown in Figure 5b. Figure 5c,d show the small changes in the profile of $I_{2}$ with an increase to $\tau_{1}$ for the studied interval of values of $\tau_{1}$ (at the same interval, we observe large changes in the profile of $I_{1}$). Figure 5c illustrates the small changes in the value of $I_{2}$, which are best visible around the peak of $I_{2}$. It is interesting that the peak of $I_{2}$ almost does not move for the studied interval of change of $\tau_{1}$.

**Figure 6:**
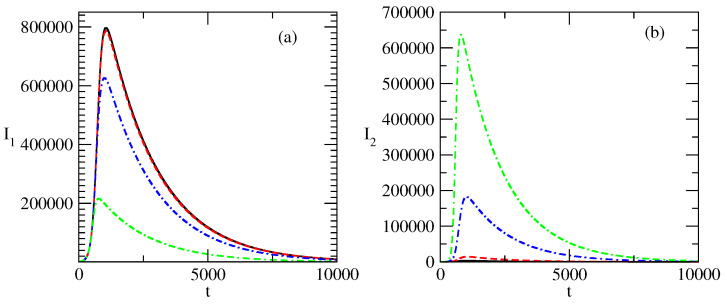
Influence of the value of $\tau_{2}$ on the profiles of $I_{1}$ and $I_{2}$ for the basic solution shown in Figure 1. (**a**) Solid line: $\tau_{1}=0.009$. Dashed line: $\tau_{1}=0.0011$. Dot-dashed line: $\tau_{2}=0.015$. Double dash-dotted line: $\tau_{2}=0.002$. (**b**) Solid line: $\tau_{1}=0.009$. Dashed line: $\tau_{1}=0.0011$. Dot-dashed line: $\tau_{2}=0.015$. Double dash-dotted line: $\tau_{2}=0.002$. The values of the parameters are as follows: $\tau_{1}=0.01$. $\rho_{1}=0.005$. $\rho_{2}=0.0006$.

Figure 6 shows the influence of the change in the transmission rate of $\tau_{2}$ on the profiles of the curves $I_{1}$ and $I_{2}$ of the infected individuals. Figure 6a shows the influence of $\tau_{2}$ on the profile of $I_{1}\left(t\right)$. The lines are thinner in order to distinguish the curves for $\tau_{2}=0.009$ and $\tau_{2}=0.011$ where the changes in the profile of $I_{1}$ are not large. The further increase in $\tau_{2}$ leads to a decrease in the peak of $I_{1}$ and to a shift of this peak to the region of earlier times. The opposite effect of the influence of the increase in the value of $\tau_{2}$ can be seen in Figure 6b. There, we observe an increase in the value of the peak of $I_{2}$ with an increasing value of $\tau_{2}$ and the shift of this peak to the region of larger times. We note that the curve of $I_{2}$ for the case $\tau_{2}=0.009$ (the solid line) is very close to the abscissa.

**Figure 7:**
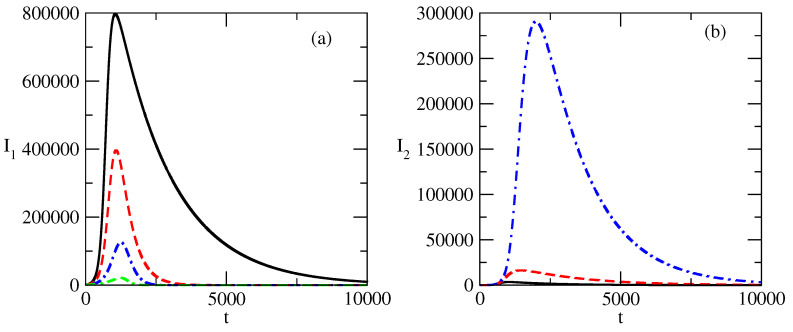
Influence of the value of $\rho_{1}$ on the profiles of $I_{1}$ and $I_{2}$ for the basic solution shown in Figure 1. (**a**) Solid line: $\rho_{1}=0.0005$. Dashed line: $\rho_{1}=0.0025$. Dot-dashed line: $\rho_{1}=0.005$. Double dash-dotted line: $\rho_{1}=0.0075$. (**b**) Solid line: $\rho_{1}=0.0005$. Dashed line: $\rho_{1}=0.0025$. Dot-dashed line: $\rho_{1}=0.005$. The values of the parameters are as follows: $\tau_{1}=0.01$. $\tau_{2}=0.009$. $\rho_{2}=0.0006$.

Figure 7 shows the influence of the recovery rate $\rho_{1}$ on the evolution of the numbers $I_{1.2}$ of infected individuals. The increase in the value of the recovery rate $\rho_{1}$ leads to a smaller peak of $I_{1}\left(t\right)$ and to a faster decrease in the value of $I_{1}$ in the course of time. Another interesting feature is that the increase in the value of $\rho_{1}$ leads to a more symmetric bell-shaped profile of $I_{1}\left(t\right)$ and to the movement of the peak of $I_{1}$ to the region of smaller values of the time *t*.

The increase in the value of $\rho_{1}$ has the opposite effect on the profile of $I_{2}$. Here, we observe the increase in the value of the peak of $I_{2}$ and a slower decrease in the value of $I_{2}$ after the peak. The peak shifts to the region of larger values of the time *t*.

**Figure 8:**
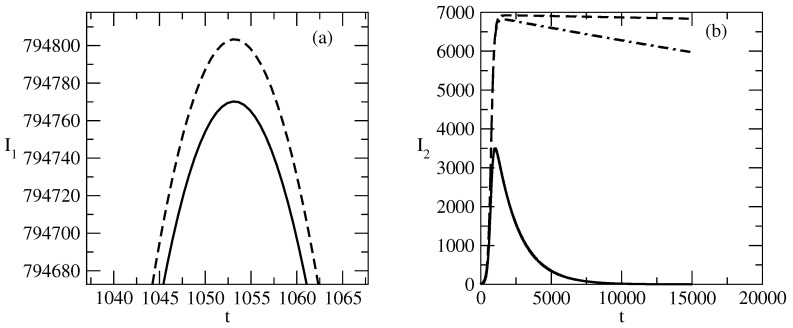
Influence of the value of $\rho_{2}$ on the profiles of $I_{1}$ and $I_{2}$ for the basic solution shown in Figure 1. (**a**) Solid line: $\rho_{2}=0.0006$. Dashed line: $\rho_{2}=0.000001$. (**b**): Solid line: $\rho_{2}=0.0006$. Dashed line: $\rho_{2}=0.000001$. Dot-dashed line: $\rho_{2}=0.00001$. The values of the parameters are as follows: $\tau_{1}=0.01$. $\tau_{2}=0.009$. $\rho_{1}=0.0005$.

Figure 8 shows the influence of the change of the value of the recovery rate $\rho_{2}$ on the profiles $I_{1,2}\left(t\right)$ of the number of infected individuals. Figure 8a shows that for the interval of studied values of $\rho_{2}$, the changes in $I_{1}\left(t\right)$ are relatively small and one observes a slight increase in the value of the peak for $I_{1}$. Figure 8b shows an interesting change in the behavior of $I_{2}\left(t\right)$ with a decrease in the value of $\rho_{2}$. At a relatively large value of $\rho_{2}$, we observe bell-shaped behavior of $I_{2}\left(t\right)$. For smaller values of $\rho_{2}$, the peak of $I_{2}$ continues to exist, but the decrease in $I_{2}$ after the peak happens very slowly.

### 4.2. $I_{1}\left(0\right)$ and $I_{2}\left(0\right)$ Have the Same Values

**Figure 9:**
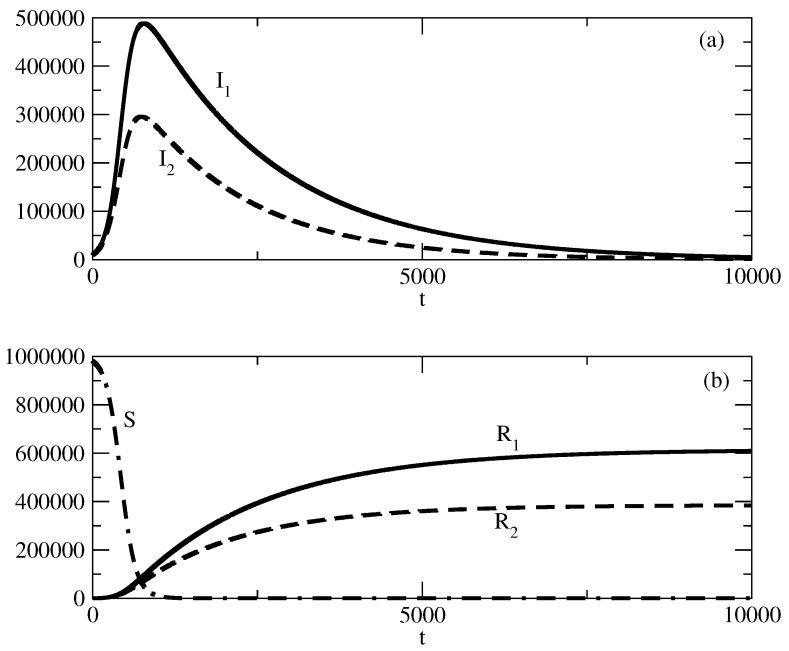
Basic solution for the studies for the case $I_{1}\left(0\right)\approx I_{2}\left(0\right)$. (**a**) $I_{1}$ (solid line) and $I_{2}$ (dashed line). (**b**) $R_{1}$ (solid line); $R_{2}$ (dashed line) and *S* (dot-dashed line). The values of the parameters are as follows: $I_{1}\left(0\right)=10,000$. $I_{2}\left(0\right)=10,000$. S(0)=980,000. $\tau_{1}=0.01$. $\tau_{2}=0.009$. $\rho_{1}=0.0005$. $\rho_{2}=0.0006$.

Figure 9 shows the basic solution for this study. For this solution, we choose $N=S+I_{1}+I_{2}=1,000,000$ and in addition, $I_{1}\left(0\right)$ is the same as $I_{2}\left(0\right)$. We observe a large wave of $I_{1}$ and a smaller wave of $I_{2}$ for the selected values of the parameters $\tau_{1,2}$ a nd $\rho_{1.2}$. The peak of the wave of $I_{2}$ is slightly earlier than the peak of the wave of $I_{1}$. Over the course of time, $I_{1,2}$ turns to 0 and the number of recovered individuals increases to *N*. We note that for the selected basic wave for our study, $\tau_{1}$ is slightly larger than $\tau_{2}$ and $\rho_{1}$ is slightly smaller than $\rho_{2}$.

**Figure 10:**
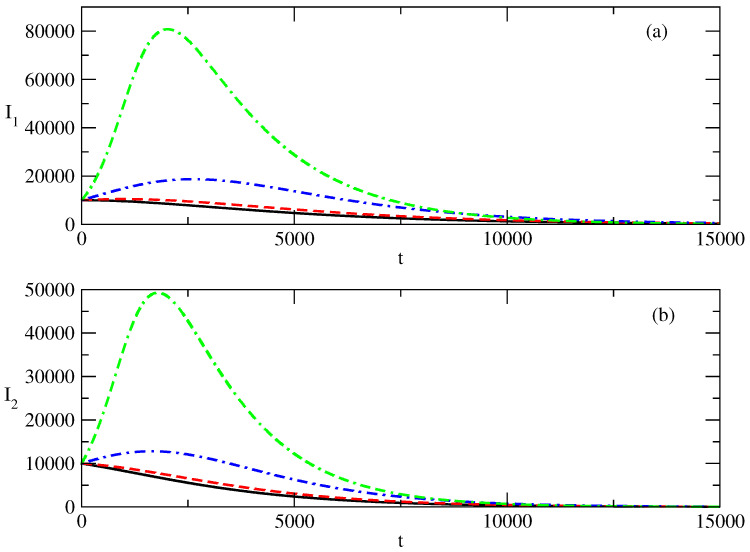
Influence of the values of S(0) on the profiles of the functions and $I_{2}\left(t\right)$. (**a**) $I_{1}$. (**b**) $I_{2}$. Solid lines: S(0)=50,000. Dashed lines S(0)=60,000. Dot-dashed lines: S(0)=100,000. Double dash-dotted lines: S(0)=250,000. The values of the parameters are as follows: $I_{1}\left(0\right)=10,000$. $I_{2}\left(0\right)=10,000$. S(0)=980,000. $\tau_{1}=0.01$. $\tau_{2}=0.009$. $\rho_{1}=0.0005$. $\rho_{2}=0.0006$.

Figure 10 shows the influence of the value of S(0) on the profiles of the functions $I_{1,2}$ of the infected individuals. We observe a monotonic decay of $I_{1}$ and $I_{2}$ for the case of relatively small values of S(0).

**Figure 11:**
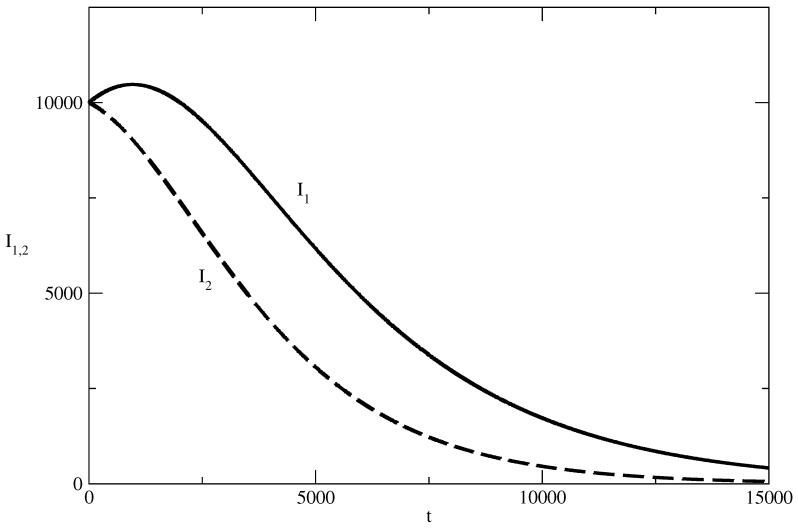
Influence of the values of S(0) on the profiles of the functions $I_{1}\left(t\right)$ and $I_{2}\left(t\right)$. S(0)=60,000. Solid line: $I_{1}\left(t\right)$. Dashed line: $I_{2}\left(t\right)$. The values of the parameters are as follows: $I_{1}\left(0\right)=10,000$. $I_{2}\left(0\right)=10,000$. S(0)=980,000. $\tau_{1}=0.01$. $\tau_{2}=0.009$. $\rho_{1}=0.0005$. $\rho_{2}=0.0006$.

Then, an interesting effect occurs, and this effect is shown in Figure 11. With an increase in the value of S(0), a peak of $I_{1}$ occurs, whereas $I_{2}$ continues to decline monotonically. When S(0) increases further, a peak also begins to form for $I_{2}$. For large values of S(0) (as can be seen in Figure 10), the bell-shaped profiles for $I_{1}$ and $I_{2}$ are well-developed.

**Figure 12:**
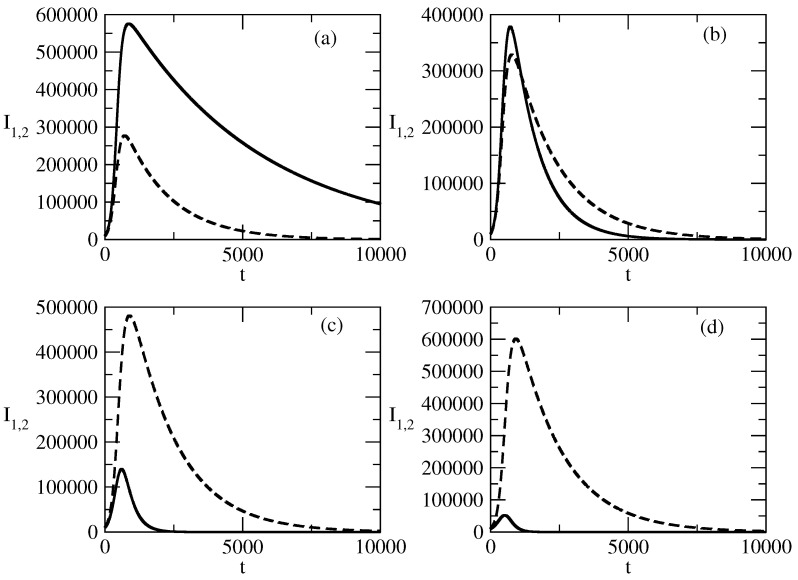
Influence of the values of $\rho_{1}$ on the profiles of the functions $I_{1}\left(t\right)$ and $I_{2}\left(t\right)$. Solid line: $I_{1}\left(t\right)$. Dashed line: $I_{2}\left(t\right)$. (**a**) $\rho_{1}=0.0002$, (**b**) $\rho_{1}=0.001$, (**c**) $\rho_{1}=0.003$, (**d**) $\rho_{1}=0.005$, The values of the parameters are as follows: $I_{1}\left(0\right)=10,000$. $I_{2}\left(0\right)=10,000$. S(0)=980,000. $\tau_{1}=0.01$. $\tau_{2}=0.009$. $\rho_{2}=0.0006$.

Figure 12 shows the influence of the value of the recovery rate $\rho_{1}$ for the population $I_{1}$ on the evolution of the curves for the infected individuals, $I_{1}$ and $I_{2}$. We observe that an increase in $\rho_{1}$ leads to a decrease in the peak of $I_{1}$. In addition, the peak shifts to the region of earlier times, and after the peak, the decay of $I_{1}$ is faster. The influence of $\rho_{1}$ on the evolution of $I_{2}$ happens in the opposite way. The increase in the value of $\rho_{1}$ leads to an increase in the value of the peak of $I_{2}\left(t\right)$. This peak becomes larger than the peak of $I_{1}$, and in addition, the peak shifts to the region of larger times. Finally, after the peak, the decay of $I_{2}$ is slower with an increasing value of $\rho_{1}$.

**Figure 13:**
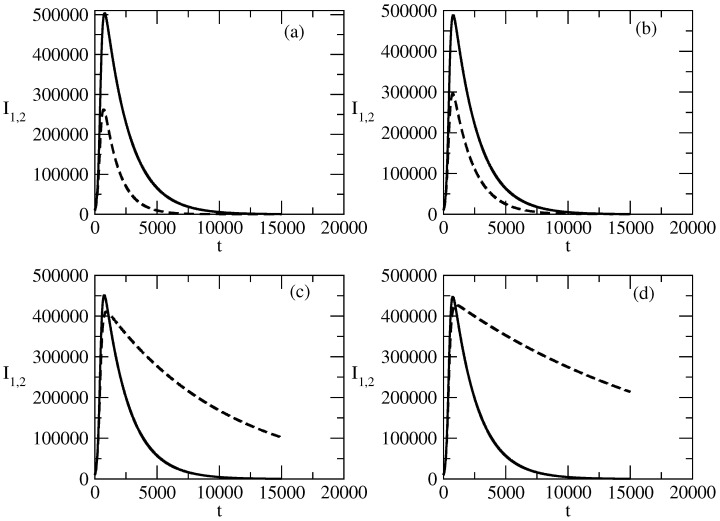
Influence of the values of $\rho_{2}$ on the profiles of the functions $I_{1}\left(t\right)$ and $I_{2}\left(t\right)$. Solid line: $I_{1}\left(t\right)$. Dashed line: $I_{2}\left(t\right)$. (**a**) $\rho_{2}=0.0008$. (**b**) $\rho_{2}=0.0006$. (**c**) $\rho_{2}=0.0001$. (**d**) $\rho_{2}=0.00005$. The values of the parameters are as follows: $I_{1}\left(0\right)=10,000$. $I_{2}\left(0\right)=10,000$. S(0)=980,000. $\tau_{1}=0.01$. $\tau_{2}=0.009$. $\rho_{1}=0.0005$.

Figure 13 shows the influence of the value of the recovery rate $\rho_{2}$ for the population $I_{2}$ on the evolution of the curves for the infected individuals, $I_{1}$ and $I_{2}$. We observe that the increase in $\rho_{2}$ leads to a slight increase in the peak of $I_{1}$ and to a slight shift of this peak to the region of larger times. The increase in the value of $\rho_{2}$ leads to a decrease in the value of the peak for $I_{2}$ and to the formation of a bell-shaped profile for $I_{2}$. The decrease in $\rho_{2}$ leads to a slow decrease in $I_{2}$ at long times and to the vanishing of the bell shape of its profile.

**Figure 14:**
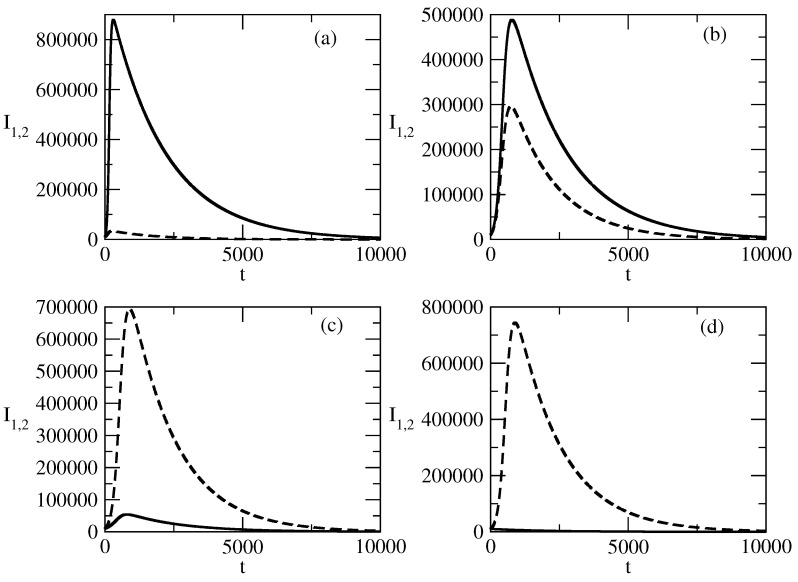
Influence of the values of $\tau_{1}$ on the profiles of the functions $I_{1}\left(t\right)$ and $I_{2}\left(t\right)$. Solid line: $I_{1}\left(t\right)$. Dashed line: $I_{2}\left(t\right)$. (**a**) $\tau_{1}=0.03$, (**b**) $\tau_{1}=0.01$, (**c**) $\tau_{1}=0.004$, (**d**) $\tau_{1}=0.0001$. The values of the parameters are as follows: $I_{1}\left(0\right)=10,000$. $I_{2}\left(0\right)=10,000$. S(0)=980,000. $\tau_{2}=0.009$. $\rho_{1}=0.0005$. $\rho_{2}=0.0006$.

Figure 14 shows the influence of the transmission rate $\tau_{1}$ on the profiles $I_{1,2}$ of the infected individuals. The decrease in the value of $\tau_{1}$ leads to a decrease in the peak of $I_{1}$ and to an increase in the value of the peak to $I_{2}$. With an increasing value of $\tau_{1}$, the decay of $I_{1}$ after the peak becomes faster and the decay of $I_{2}$ becomes slower.

**Figure 15:**
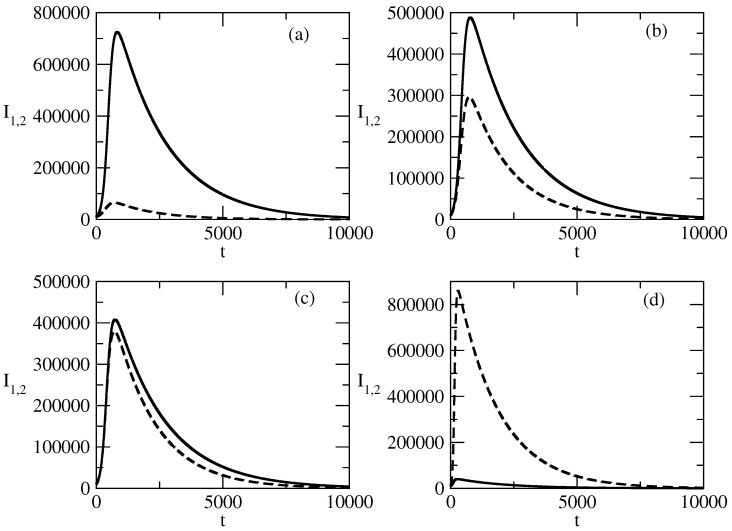
Influence of the values of $\tau_{2}$ on the profiles of the functions $I_{1}\left(t\right)$ and $I_{2}\left(t\right)$. Solid line: $I_{1}\left(t\right)$. Dashed line: $I_{2}\left(t\right)$. (**a**) $\tau_{2}=0.005$, (**b**) $\tau_{2}=0.009$, (**c**) $\tau_{2}=0.01$, (**d**) $\tau_{2}=0.03$. The values of the parameters are as follows: $I_{1}\left(0\right)=10,000$. $I_{2}\left(0\right)=10,000$. S(0)=980,000. $\tau_{1}=0.01$. $\rho_{1}=0.0005$. $\rho_{2}=0.0006$.

Figure 15 shows the influence of the transmission rate $\tau_{2}$ on the profiles $I_{1,2}$ of the infected individuals. The increase in the value of $\tau_{2}$ leads to an increase in the value of the peak of $I_{2}$ and to a decrease in the value of the peak of $I_{1}$. The decay of $I_{2}$ after the peak becomes slower with an increasing value of $\tau_{2}$, and at the same time, $I_{1}$ decays faster.

## 5. Discussion of the Obtained Exact Analytical Solutions of the Studied Chain of Equations from the Point of View of Modeling of Waves of Popularity

We will use the SIIRR model to model the spread of waves of popularity.

1. We consider the popularity change as a process of infecting individuals of a population with a possibility of a recovery (losing interest in the popular person/idea).
2. The popularity of something (person or idea) is measured by the number of infected individuals (individuals who have an opinion about the corresponding person or idea).
3. We distinguish between two kinds of popularity:
  (a) Positive popularity;
  (b) Negative popularity.
  In the case of positive popularity, the “infected” have a positive opinion about the person or towards the idea. In the case of negative popularity, the “infected” have a negative opinion about the person or towards the idea.
4. The positive popularity and the negative popularity coexist. They can be considered as two different infections with a specific feature.
5. The specific feature is that if one is infected by positive popularity, this person cannot be infected by negative popularity at the same time.
6. Recovering from the infection with positive or negative popularity can also occur. In this case, the corresponding person becomes indifferent to the popular person or the popular idea.
7. Thus, in the SIIRR model, we have suspected individuals *S* which can be infected by the positive or negative popularity of an individual (or group of individuals) or towards an idea (or system of ideas).
8. The two kinds of infection are positive popularity ($I_{1}$ infected individuals) and negative popularity ($I_{2}$ infected individuals).
9. There are also two groups of recovered individuals. $R_{1}$ is the group of individuals who recovered from positive popularity and $R_{2}$ is the group of individuals who recovered from negative popularity. The assumption is that recovered individuals cannot be infected further: they become indifferent to the person or towards the idea or system of ideas.

The process of spreading popularity in a population (represented by $I_{1}$ and $I_{2}$) is modeled by the SIIRR model (3). The model describes the evolution of waves of positive or negative popularity which have a maximum value (peaks of positive or negative popularity) and then $I_{1}$ and $I_{2}$ tend to 0: the popularity vanishes regardless of whether it is positive or negative.

In Figure 1, we consider the basic solution for the case $I_{1}\left(0\right)>>I_{2}\left(0\right)$. We note again that because of the symmetry of the model equations, we will obtain similar results in the case $I_{2}\left(0\right)>>I(0)$. From the point of view of popularity waves, we observe the following in Figure 1.

1. We associate $I_{1}$ with positive popularity and $I_{2}$ with negative popularity. We observe a large wave of positive popularity and a much smaller wave of negative popularity.
2. The peak of the wave of negative popularity occurs slightly earlier than the peak of the wave of positive popularity.
3. For the discussed specific case, the corresponding person or idea (group of individuals or groups of ideas) is (are) much more popular than unpopular.
4. Over the course of time, popularity and unpopularity tend to 0 and the number of indifferent individuals increases to *N*. The system is ready to deal with the next popular individuals or ideas.
5. Thus, popularity occurs, rises, falls, and vanishes.

Figure 2 shows the influence of the initial number of susceptible individuals S(0) on the evolution of positive and negative popularity. S(0) represents the part of the total population which can be affected by the waves of positive and negative popularity. As $S\left(0\right)=N-I_{1}\left(0\right)-I_{2}\left(0\right)$, it appears to be an important factor if S(0) is large enough or it is not large enough. We observe fascinating behavior of $I_{1,2}$ with respect to the last factor.

1. For small values of the initial number of the susceptible individuals S(0), the positive and negative popularity of the individual/group of individuals or of the idea/group of ideas decreases monotonically from its initial value at t=0. This means that it is very important to have a much larger number of individuals which can be affected by popularity in comparison to the initial numbers $I_{1,2}\left(0\right)$ connected to positive and negative popularity. We call this phenomenon “suppression of popularity”.
2. If the number of susceptible individuals is large enough, there is a change in the behavior of the system. In this case, classical bell-shaped curves of popularity (positive and negative) occur. One observes an increase and peak of the positive and negative popularity, and then the popularity decreases to 0. This is the “effect of forgetting”: society “forgets” the popular person/group of persons or the popular idea/group of ideas.
3. Different times of occurrence of the peaks of positive and negative popularity can be present. We call this the “effect of the delay of a peak”. In the discussed case, the peak of negative popularity occurs earlier. In this case, for some time, the positive popularity increases and the negative popularity decreases. We call this time period the “window of dominance”. Of course, for other values of the parameters, we could have a peak of positive popularity before a peak of negative popularity. Then, we have another “window of dominance”: a time window in which the positive popularity decreases and the negative popularity still increases.
4. Finally, in Figure 2b,d, we observe that the positive popularity can peak very fast, but then the decrease is also very fast. In contrast, the negative popularity can peak slowly, but then the decay of this popularity is also very slow. We call this effect “short term win–long term loss”. For other values of the parameters, we could have the opposite situation: short-term win for the negative popularity, which leads to its long-term loss.

Figure 3 gives information for the case where the initial value of positive popularity increases and the initial value of the negative popularity does not increase. In this case, the positive popularity $I_{1}\left(t\right)$ reaches its maximum faster, and after this, we observe a faster decrease in positive popularity in comparison to the case of the smaller $I_{1}\left(0\right)$.

1. A larger initial value of positive popularity leads to a larger peak.
2. In addition, the larger initial value of positive popularity suppresses the negative popularity: the peak value of negative popularity decreases and occurs earlier in time.
3. Thus, the goal of an image-maker can be to increase the initial positive popularity of the corresponding individual/group of individuals or idea/group of ideas. In such a way, the coexisting wave of negative popularity will be suppressed.
4. But this has a price: the peak of positive popularity will occur earlier and then one will observe a faster decrease in this popularity.

Figure 4 shows the influence of the increased value of the initial negative popularity $I_{2}\left(0\right)$ on the evolution of the positive popularity $I_{1}\left(t\right)$ and negative popularity $I_{2}\left(t\right)$.

1. The increase in the initial value of the negative popularity leads to a sharp decrease in the positive popularity, as can be seen in Figure 4a.
2. At the same time, the larger initial value of negative popularity can lead to a large increase in this popularity—Figure 4b.
3. Thus, we have an important mechanism for the control of positive popularity: we just need to have a large enough value of the corresponding negative popularity.

Figure 5 shows the influence of the value of the transmission rate $\tau_{1}$ on the evolution of the positive popularity $I_{1}\left(t\right)$ and negative popularity $I_{2}\left(t\right)$. A larger transmission rate $\tau_{1}$ means that the positive popularity spreads easily.

1. We observe that for small values of $\tau_{1}$, the positive popularity can decrease and the occurrence of a peak in this popularity happens for values of $\tau_{1}$ over a certain threshold.
2. Further, an increase in $\tau_{1}$ can lead to a jump of positive popularity.
3. At the same time, the negative popularity $I_{2}$ can remain almost unchanged, as can be seen in Figure 5c,d.

Figure 6 shows the influence of the transmission rate $\tau_{2}$ on the evolution of the positive popularity $I_{1}\left(t\right)$ and negative popularity $I_{2}\left(t\right)$.

1. A larger transmission rate $\tau_{2}$ means that the negative popularity spreads easily. This influences the positive popularity: the easier spread of negative popularity inhibits the positive popularity. The peak of positive popularity becomes smaller and it occurs earlier.
2. Opposite to this, the larger transmission rate for the negative popularity leads to larger negative popularity: the peak of the negative popularity becomes larger and this peak occurs later in time.
3. Thus, if we want to suppress positive popularity and increase negative popularity, we have to increase the transmission rate of the negative popularity. This decreases the peak of positive popularity and increases the peak of the negative popularity.
4. In addition, the positive popularity will last less (as its peaks will move to an earlier time) and the negative popularity will last longer (as its peak will move to the region of larger times).

Figure 7 shows the influence of the change of the value of the recovery rate $\rho_{1}$ of the population, forming positive popularity $I_{1}$. The larger value of the recovery rate $\rho_{1}$ means that the individuals become at a larger rate indifferent to the positive popularity of an individual/group of individuals or to an idea/group of ideas.

1. The increase in the value of the recovery rate $\rho_{1}$ negatively influences the positive popularity $I_{2}\left(t\right)$. The value of the peak of this popularity can fall dramatically. In addition, the smaller peak of popularity shifts to the region of smaller times. This means that the positive popularity increases with larger difficulties and decays faster in comparison to the case when the value of the recovery rate $\rho_{1}$ is smaller.
2. Figure 7b shows the opposite effect on the increase in the value of the recovery rate $\rho_{1}$ on the evolution of the negative popularity $I_{2}$. The increase in the value of the recovery rate for positive popularity leads to an increase in negative popularity. The peak of the negative popularity can become very large and, in addition, the peak moves to the region of larger times. This means that the negative popularity increases faster and lasts longer.

Figure 8 shows the influence of the change in the value of the recovery rate $\rho_{2}$ of the negative popularity.

1. Figure 8a shows that there is an interval of values for $\rho_{2}$ where a change in the value of $\rho_{2}$ leads to small changes in positive popularity and this popularity is almost not affected.
2. An interesting influence of the change in the value of the recovery rate for negative popularity on the evolution of the negative popularity is shown in Figure 8b. Here we observe a bell-shaped behavior of the curve of positive popularity, and the decrease in the value of $\rho_{2}$ leads to reaching a peak value followed by a very slow decrease in the positive popularity.

In Figure 9, we present the basic studied case for the same values of initial positive popularity and negative popularity ($I_{1}\left(0\right)=I_{2}\left(0\right)=10,000$). In this case, we have a significant initial number of infected individuals and there are two large waves of positive and negative popularity.

1. The peak of the wave of negative popularity is slightly earlier than the peak of the wave of positive popularity.
2. We observe polarization of society with respect to the corresponding person or idea.
3. Despite this polarization, in the course of time, popularity decreases and then tends to 0.
4. Figure 9b shows the corresponding evolution of the susceptible and recovered individuals. The number of susceptible individuals decreases fast, and then society is divided to groups of infected and indifferent individuals.
5. For a short time, popularity prevails over indifference, but as time advances, society loses its interest in the corresponding person or idea.

Figure 10 and Figure 11 present the influence of the initial number of individuals susceptible to the positive or negative popularity of the person(s) or idea(s) on the evolution of the positive and negative popularity over time.

1. In the case of a small number of individuals susceptible to the corresponding popularity, we again observe the effect of suppression: the positive and negative popularity decrease monotonically with increasing time.
2. Then, in Figure 11, we observe the “effect of the single peak”. Here, the negative popularity again decreases monotonically, and the positive popularity has a peak before the beginning of its decay. Of course, for different values of the parameters, we can have monotonous decay for positive popularity and a peak for negative popularity.
3. With a further increase in the initial number of individuals susceptible to “popularity infection” the curves for positive and negative popularity develop peaks and then there is a decay. We observe again “the effects of forgetting”, “the effect of delay of the peak” and the “windows of dominance”.

Figure 12 shows the influence of the value of the recovery rate $\rho_{1}$ for the positive popularity on the evolution of positive popularity and negative popularity.

1. The increase in the recovery rate for positive popularity negatively influences the positive popularity and positively influences the negative popularity. We observe that the increase in $\rho_{1}$ leads to a decrease in the peak of the positive popularity.
2. In addition, this peak shifts to the region of earlier times, which means that the positive popularity begins to decrease earlier.
3. The second negative effect of the increasing value of $\rho_{1}$ is that the positive popularity decreases faster at the times after the peak.
4. On the contrary, the increase in the value of the recovery rate for positive popularity positively affects the negative popularity $I_{2}$.
5. The positive effects are that the peak value of the negative popularity becomes larger. In addition, the peak shifts to the region of larger times, i.e, the wave of negative popularity increases faster and lasts longer.
6. Finally, the decay of this wave becomes slow.

Figure 13 shows the influence of the value of the recovery rate $\rho_{2}$ for negative popularity on the evolution of positive popularity and negative popularity.

1. We observe that the increase in the recovery rate for negative popularity leads to a slight increase in the peak of $I_{1}$ and to a slight shift of this peak to the region of larger times.
2. The decrease in $\rho_{2}$ leads to an increase in the value of the peak of $I_{2}$ and to a shift of this peak to the region of larger times.
3. In addition, the decay of $I_{2}$ after the peak is slower.

Figure 14 shows the influence of the transmission rate $\tau_{1}$ of positive popularity on the positive and negative popularity in the studied system.

1. The decrease in the value of the transmission rate $\tau_{1}$ leads to a decrease in the peak of positive popularity and to increase in the value of the peak for negative popularity.
2. With an increasing value of $\tau_{1}$, the decay of the positive popularity after the peak becomes faster and the decay of the negative popularity happens more slowly.
3. Thus, the decrease in the transmission rate of positive popularity negatively influences the positive popularity and positively influences the negative popularity.

Figure 15 shows the influence of the transmission rate $\tau_{2}$ for the negative popularity on the positive and negative popularity in the system.

1. The increase in the value of $\tau_{2}$ leads to an increase in the value of the peak of negative popularity and to a decrease in the value of the peak of positive popularity.
2. The decay of negative popularity after the peak happens more slowly with an increasing value of $\tau_{2}$ and, at the same time, the positive popularity decays faster.

## 6. Concluding Remarks

A life event led us to the development of this mathematical theory. A colleague of ours (a chemist) made a political career and became Prime Minister of Bulgaria. We observed the formation of two groups of individuals with different opinions about this person. The first group had the opinion that the Prime Minister is skilled and very good for this job. The second group had the opposite opinion, and this negativism was aired by a famous politician as “Niki will not travel anymore” (the first name of the Prime Minister is Nikolay). We observed how the number of members of the two groups increased in the course of time and then people started to become indifferent and the number of members of the groups started to decrease (especially after our colleague was not Prime Minister anymore). Thus, we came to the idea that waves of popularity are present in society and these waves are waves of positive and negative popularity. The mathematical theory of such waves can be built on the basis of a model of the spreading of two viruses in a population. And the simplest model is the SIIRR model discussed in this article.

The SIIRR model contains as a specific case the classic SIR model of epidemic spread. The infection studied in the SIIRR model has two specific features:

1. If an individual is infected by one of the viruses, then this individual cannot be infected by the second virus;
2. The individuals who have recovered from an infection cannot be infected again.

This version of the SIIRR model is designed to study popularity waves. One can obtain an analytical solution of the SIIRR model for specific values of the model parameters. This solution describes the process of decay of those infected by one of the infections and the bell-shaped curve for those infected by the second infection. From the point of view of popularity waves, the analytical solution describes a steady decrease in one kind of popularity and bell-shaped behavior of the other kind of popularity.

In addition, we conduct a numerical investigation of the model nonlinear differential equations. We note that the numerical study also leads to specific cases where one of the waves of popularity decreases steadily, whereas the wave of the opposite popularity has bell-shaped behavior. In addition, the numerical study shows that the parameters of the model influence the popularity waves. If one wants to increase the peak of positive popularity and decrease the peak of negative popularity, the value of the transmission rate $\tau_{1}$ must be increased. The same effect can be achieved by increasing the recovery rate $\rho_{2}$ of the negative popularity. The increase in the transmission rate for the negative popularity $\tau_{2}$ leads to the opposite effect. This can also be achieved by increasing the recovery rate $\rho_{1}$ of positive popularity. The changes in these rates also change the position of the peaks of the positive and negative popularity at time. The initial number of suspected individuals S(0) also influences the evolution of positive and negative popularity very much. Thus, by appropriate selection of the transmission and recovery rates, one can achieve a time window of dominance of one kind of popularity over the other kind of popularity.

The numerical results show the existence of different effects connected to evolution of the numbers of infected individuals in the course of time. We describe such effects in the text:

1. Arising of bell-shaped profiles of the numbers of supporters of positive popularity and negative popularity;
2. Suppression of popularity;
3. Faster increase–faster decrease effect for the peaks of the bell-shaped profiles;
4. Shift of the peak of popularity in time;
5. Effect of forgetting;
6. Window of dominance;
7. Short-term win–long-term loss effect;
8. The effect of the single peak where one kind of popularity decreases steadily and the other kind of popularity increases, then has a peak and, after that, begins to decrease.

Finally, we note that the discussed model can be considered as a model of popularity evolution not only of single individuals but also of groups of individuals. Moreover, the theory can be used to model the popularity of an idea or a group of ideas and even to model the popularity of entire ideologies. The mathematical theory discussed above will lead to numerous models of popularity based on the models of spread of diseases. We will present other results on this elsewhere.

## Data Availability

No new data were created or analyzed in this study. Data sharing is not applicable to this article.
